# Cortical auditory evoked potentials and hemispheric specialization of speech in individuals with learning disability and healthy controls: A preliminary study

**DOI:** 10.12688/f1000research.17029.1

**Published:** 2018-12-17

**Authors:** Mayur Bhat, Hari Prakash Palaniswamy, Arivudai Nambi Pichaimuthu, Nitha Thomas

**Affiliations:** 1 Department of Speech and Hearing, School of Allied Health Sciences, Manipal, Karnataka, 576104, India; 2 Department of Speech and Hearing, Kasturba Medical College Hospital, Mangalore, Karnataka, 575003, India; 3 Department of Clinical Psychology, School of Allied Health Sciences, Manipal, Karnataka, 576104, India

**Keywords:** CAEPs, hemispheric asymmetry, dichotic listening, learning disability

## Abstract

**Background: **Dichotic listening (DL) technique is a behavioral non-invasive tool which is used in studying hemispheric lateralization. Previous studies using behavioral DL have hypothesized that individuals with learning disabilities (LD) exhibit a lack of cortical specialization for processing speech stimulus. However, there is no event related potential (ERP) evidence, hence the main objective of the study is to explore hemispheric asymmetry using cortical auditory evoked potential (CAEPs) in normal hearing adults and also to compare the same in children with LD and healthy controls.

**Methods: **CAEPs were recorded in 16 normal hearing young adults, eight right-handed children with LD and their age matched controls. Two stop constants (/Pa/ – voiceless, bilabial, stop: /Ta/ - voiceless, alveolar, stop) were chosen for this experiment and presented in each ear and dichotically in two different orders (/pa-ta/, /ta-pa/). ERPs were processed using a standard pipeline, and electrodes readings over the left and right hemispheres were averaged to create left and right regions of interest (ROI). The CAEPs were analyzed for mean amplitude and peak latency of P1-N1-P2 components.

**Results: **The current study results suggest no statistically significant difference between the two stimulus in monaural condition and absence of order effect in dichotic condition. In healthy controls the CAEP latencies were shorter over the left hemisphere in both monaural and dichotic conditions in adults and control children. However, it was very evident that such a difference was lacking in children with LD.

**Conclusions:** Hemispheric asymmetry can be detected using CAEPs for speech stimulus. The measures are consistent and void of stimulus or order effect. Taken together, the findings of current study, both monaural and dichotic condition illustrates the hemispheric differences in processing speech stimuli in normal hearers. Absence of latency differences between hemispheres in children with LD indicate a lack of hemispheric asymmetry.

## Introduction

The human brain is comprised of two hemispheres, and both hemispheres differ from each other in terms of anatomy as well as physiology. Among the two hemispheres, one is more active and demonstrates superior performance on specific tasks. This phenomenon is referred to as brain dominance or hemispheric asymmetry. This brain dominance seems to be related to the handedness of the person. In humans, brain dominance or asymmetry seems to be established early in fetal development. Dichotic listening (DL) is one of the conventional methods to study this cerebral dominance effect ( Ahonniska *et al*., 1993). In this test, two different auditory signals are presented to two ears independently, and the listeners are expected to recognize the signals presented to both ears. The DL test is sensitive to hemisphere differences to specific sounds ( Brancucci *et al*., 2008). The principle of this test is that speech is lateralized to the left hemisphere ( Tervaniemi & Hugdahl, 2003), resulting in the individual preferring to repeat the stimulus presented to the right ear more often than the left ear. The vice versa is true when it comes to non-speech stimulus ( Brancucci *et al*., 2008). These effects are termed as right ear advantage (REA), and left ear advantage (LEA) respectively and highly correlates with the Wada-test ( Hugdahl *et al*., 1997). Since, it is a simple, effective and non-invasive equivalent of the Wada-test, it has been widely used in the assessment of various clinical populations. Therefore the DL technique has been used widely as a measure of cortical processing and auditory perception for several decades ( Hugdahl *et al*., 1997).

DL technique has been found to be useful in studying language lateralization in children with early focal brain damage ( Brizzolara *et al*., 2002; Carlsson *et al*., 1992; Chilosi *et al*., 2005; Isaacs *et al*., 1996), aphasia ( Bavosi & Rupp, 1984; Johnson *et al*., 1977; Johnson *et al*., 1978; Pettit & Noll, 1979; Selnes *et al*., 1983), and stuttering ( Brady & Berson, 1975; Blood & Blood, 1986; Curry & Gregory, 1969; Foundas *et al*., 2004; Gruber & Powell, 1974; Robb *et al*., 2013; Slorach & Noehr, 1973; Strub *et al*., 1987). The DL test is particularly useful in the assessment of children with learning disability. The DL tests have been used to reveal cerebral dominance deficits ( van den Noort *et al*., 2008), subtypes of children with dyslexia ( Cohen *et al*., 1992), developmental changes in language lateralization ( Porter & Berlin, 1975), and bilateral hemispheric processing deficits ( Obrzut & Mahoney, 2011) in children with LD.

Traditionally, DL has been assessed using behavioral methods. However, electrophysiological methods, especially cortical auditory evoked potentials (CAEPs), would help in understanding the neurophysiology of dichotic listening. Since the left hemisphere is dominant for speech and language function, CAEPs in DL is evidenced by larger amplitudes and shorter latencies over the left hemisphere ( Bayazit *et al*., 2009; Eichele *et al*., 2005; Friedrich *et al*., 2017; Haaland, 1974; Morrell & Salamy, 1971). However, these studies have either focused on latency ( Eichele *et al*., 2005) or amplitude ( Haaland, 1974; Morrell & Salamy, 1971), but not both. Hence, the complete cortical dynamics underlying processing is not yet known. Analyzing both these measures will provide information on the strength of cortical activation, as well as the efficiency of neural conduction.

Dichotic study involves the presentation of one stimulus to right ear and other stimulus to left ear. Previous studies have reported cortical changes using dichotic, but have not explored stimulus effect or order effect ( Bayazit *et al*., 2009; Eichele *et al*., 2005; Friedrich *et al*., 2017; Haaland, 1974; Morrell & Salamy, 1971). The physiological responses elicited using different stimulus may differentially influence evoked ERPs, since these are obligatory responses to external stimuli. It is important to rule out stimulus specific effects before commenting on cortical asymmetry using this paradigm. Hence, the current study aimed at studying the stimulus effect in monotic (/pa/ vs. /ta/) and order effect in dichotic condition (/pa-ta/ vs. /ta-pa/). The study also aimed at comparison of monaural vs. dichotic processing differences in the same individual. This will provide important insight on how cortical processing of dichotic listening differs from that of monaural listening.

 Previous studies using behavioral DL have hypothesized that individuals with LD exhibit a lack of cortical specialization for processing speech stimulus. To date there is no literature evidence for this using ERPs. A handful of studies have utilized CAEPs to study DL in children with LD, and revealed a comparable amplitude between hemispheres indicating decreased cortical asymmetry for speech stimulus ( Brunswick & Rippon, 1994). Hence studying cortical processing of dichotic listening using ERPs will further validate these findings. In this view, there is a definite need for a study to establish the monaural and dichotic auditory processing differences as reflected by CEAPs in healthy individuals and those with LD.

## Methods

The study was carried out at the Department of Speech & Hearing, School of Allied Health Science, and Manipal. The study began on 1 ^st^ August 2016 and continued till 20 ^th^ August 2017. The study protocol was approved by Institutional Ethics Committee (IEC), Kasturba Hospital, Manipal (IEC 460/2016).

### Participants

This study was a prospective observational study where 16 normal young adults (18–25 years), eight normal learning right-handed children (7–15 years) for the control group and eight right-handed individuals with LD (7–15 years) were recruited. Healthy volunteers were either students at the School of Allied Health Sciences, who were recruited through advertisement through notice board or members of the public who visited the department. They had the study explained to them and were recruited if interested in participating. All the Volunteers were provided with participant information sheet which had complete details of the study. Written informed consent was taken from all interested individuals prior to participation in the study.

All the participants were screened for the presence of hearing loss (Pure Tone Audiometry done using duly calibrated Madsen Astera (American National Standard Institute S3.43-1996) should be <15dBHL (decibels Hearing Level) for both air conduction and bone conduction tests, and middle ear dysfunction (Tympstar middle ear analyzer, Grason-Stadler Inc., MN, USA). All the tests were carried out by investigators (audiologist) at Dept. of Speech and Hearing, School of Allied Health Sciences). Individuals who were diagnosed with a learning disability at the Department of Psychology, SOAHS, Manipal University, Manipal and concented to participate in the study were included in the experimental group. Edinburg’s handedness inventory ( Oldfield, 1971) was administered to the participants, and only right-handed individuals were selected for the study because handedness is considered as a major variable that affects cortical asymmetry. ( Delorme & Makeig, 2004)

### Stimulus preparation

 Two speech sounds (/Pa/ – voiceless, bilabial, stop: /Ta/ - voiceless, alveolar, stop,), were selected as stimulus to elicit ERP. Syllables (/Pa/ and /Ta/) were used as a stimulus for both monaural and dichotic paradigms. Also, similar stimuli has been shown to be effective in eliciting LLR and used in studying cerebral asymmetry in the literature ( Lawson & Gaillard, 1981). The above syllables were recorded using a standard microphone kept at a 6cm distance from the mouth (Extended data ( Palaniswamy, 2018b)). A normal native Kannada speaker was asked to produce these two syllables with normal intensity and normal intonation. The dichotic stimulus was prepared using Adobe Audition version 1.0, where stimulus /pa/ was stored in the right channel and /ta/ was stored in the left channel to create a single / pa-ta/ dichotic stimuli, and vice-versa to create /ta-pa/ dichotic stimuli. Duration of the stimulus was trimmed so that both the stimulus had the same duration.

### Stimulus presentation and LLR recording

All the measurements were carried out in an acoustically treated room. The ‘SOUND’ module of Stim system (Version 2) was used for stimulus presentation with inserts at an intensity of 70dBSPL. CEAPs were recorded using the ‘Acquire’ module of the SynAmps2 amplifier (Compumedics NeuroScan, Abbotsford, Australia). A 32 channel electrode cap used with combined mastoid as reference. Impedance at all electrode sites was maintained below 5k Ohms. Raw EEG recording were acquired with a bandpass filters set between 0 and 100Hz with a sampling rate of 1000/sec. The obtained EEGs were analyzed offline using a filter from 1 – 30 Hz, and artifact rejection was also be done offline ( EEGLab version 13_6_5b ( Delorme & Makeig, 2004)).

### Conditions

The stimulus was presented in 3 conditions.

1. A monaural condition in which stimulus (/pa/ and /ta/) was presented to the right ear only
2. A monaural condition in which stimulus (/pa/ and /ta/) was presented to the left ear only.
  In both, the monaural conditions patient will be asked to watch a silent movie and ignore the stimulus presented to the ear.
3. Dichotic passive attention condition in which the stimulus (/pa-ta/ and /ta-pa/) was presented to both ears simultaneously and the patient will be asked not to pay attention to the stimulus.

### Stimulus effect and order effect

The current study used two stimuli (/pa/ and /ta/) to record CAEPs. In monaural condition, two stimuli were presented to each ear one at a time to check whether the stimulusaffects CAEPs. Similarly, in dichotic condition, /pa/-/ta/ was presented to the right and left rear respectively, and the reverse of it, i.e., /ta/-/pa/ was used to check if reversal of stimulus order had any effect on CAEPs. Thus a total of 6 conditions were obtained from a single participant.

### Data analysis

Raw EEG data were imported to EEGLab version 13_6_5b ( Delorme & Makeig, 2004), a free software commonly used for analyzing EEG/ERP signals offline, which runs on MATLAB (2010a). The following preprocessing steps were done serially on each data to obtain a final average waveform. After editing channel locations (BESA 4 shell dipfit spherical model) bad channels and bad blocks were visually inspected and interpolated using spherical interpolation method in the command line in MATLAB. The data was then subjected to high pass filtering with a cut-off frequency of 1kHz. Bin based epochs were extracted using ERPLAB version 6.14 ( Luck, 2014) between -200 to 800ms timelocked to stimulus onset, and then were baseline corrected for the prestimulus duration (-200 to 0ms). Independent component analysis (ICA) was done to decompose multivariate ERP waveform into their subcomponent based on their source using the ‘runica’ command in EEGLAB, then analysed using MARA 1.1 (Multiple Artefact Rejection Algorithm) ( Winkler *et al*., 2011) which automatically removes the components with artifacts based on several parameters. Post artifact rejection, the waveforms were low pass filtered with a cutoff frequency of 30Hz and then rereferrened to common average. All the epochs were averaged in ERPLAB.

### The region of interest (ROI)

A region of interest (ROI) is one of the prescribed methods of analyzing ERPs where few neighboring electrodes that represents a particular anatomical area for a specific purpose are selected for analysis rather than a single electrode. The basis of this type of analysis is mainly on certain assumptions. The first reason is to explore one's data. It is often useful to see the activity in areas of interest plotted for each condition or plotted against other variables. The second reason is to control Type I error by restricting the number of statistical tests to a few ROIs. The third reason is to limit testing to a specific brain region that is defined functionally by some information ( Poldrack, 2007).

In the current study, two 2 ROIs with three electrodes in each hemisphere were selected, here in after synonymously referred to as right and left hemisphere electrodes. Left ROI was an average of 3 electrodes (C3, FC3, and CP3), and the right hemisphere ROI was an average of homologs of the these three electrodes (C4, FC4, CP4). For example, the latency of N1 component from C3, FC3, and CP3 are 108ms, 110ms, 112ms respectively; then the left hemispheric ROI is 110ms. The right and left hemispheric ROI were obtained for all the three conditions. Hence monaural right condition included monaural right ear right hemisphere electrodes (MonoR RH), and monaural right ear left hemisphere electrodes (MonoR LH). Monaural left conditions included monaural left ear right hemisphere (MonoL RH) electrodes, and monaural left ear left hemisphere electrodes (MonoL LH). Dichotic conditions included dichotic right hemisphere electrodes (DI RH) and dichotic left hemisphere electrodes (DI LH).

Grand mean average waveform across participants was used as a reference to decide the latency range of measurement. In the current study, for all the conditions across groups, P1 mean amplitude and peak latency was measured between 40 to 80 msec. Similarly, 90 to 140 msec, and 170 to 220 msec windows were used for N1 and P2 respectively. These mean amplitudes and peak latency measures for right and left ROIs were automatically measured using the measurement toolbox of ERPLAB for each participant and the output was written in .txt format then later exported to MS Excel 2016 and SPSS version 15 (SPSS Inc., Chicago)

## Results

All the data were first tested for normality using Shapiro-Wilk’s test, and the results showed that the latencies and amplitudes of all the components were normally distributed.

Since the use of two stimuli is inevitable in dichotic listening, it was a must to rule out any stimulus effect on CAEPs. In monaural condition, these two stimuli (/pa/ vs. /ta/) did not result in significant latency or amplitude difference in both right and left ear ( Table 1). Similarly, in dichotic condition comparison of two stimuli in a different order (/pa-ta/ vs. /ta-pa/) also did not lead to any significant difference ( Table 1) which ruled out order effect. Given this, data was combined across stimuli for rest of the analysis. ANOVA with repeated measures (3×2×3) was carried out to check for main effect and interaction effect.

**Table 1.** Statistics of Stimulus effect for different components in all the three groups.

Component	Condition	Measures	Group		
			Normal Adults	Normal Children	LD Children
P1	Dichotic	Latency	t(15)=1.809, p=0.09	t(7)=-0.469, p=0.635	t(7)=0.649, p=0.537
		Amplitude	t(15)= 0.289, p=0.706	t(7)=0.998, p=0.315	t(7)=-0.087, p=0.931
	Monaural Left	Latency	t(15)=1.363, p=0.193	t(7)= -0.412, p=0.692	t(7)=-0.239, p=0.818
		Amplitude	t(15)=-0.044, p=0.965	t(7)= -0.447, p=0.668	t(7)= 0.470, p=0.652
	Monaural Right	Latency	t(15)=-0.083, p=0.935	t(7)= -0.057, p=0.961	t(7)= -0.168, p=0.872
		Amplitude	t(15)= 0.905, p=0.380	t(7)=0.030, p=0.977	t(7)= -0.302, p=0.772
N1	Dichotic	Latency	t(15)= 0.845, p=0.411	t(7)=0.201, p=0.866	t(7)= -0.138, p=0.898
		Amplitude	t(15)=1.835, p=0.086	t(7)=0.164, p=0.874	t(7)= -0.603, p=0.567
	Monaural Left	Latency	t(15)= 1.523, p=0.149	t(7)=-0.552, p=0.598	t(7)= 0.358, p=0.737
		Amplitude	t(15)=1.051, p=0.318	t(7)= -0.052, p=0.960	t(7)=0.763, p=0.218
	Monaural Right	Latency	t(15)= -1.048, p=0.179	t(7)=0.492, p=0.638	t(7)=-0.646, p=0.539
		Amplitude	t(15)= -1.364, p=0.193	t(7)=-0.889, p=0.404	t(7)=-0.068, p=0.984
P2	Dichotic	Latency	t(15)=-0.623, p=0.543	t(7)=0.567, p=0.588	t(7)=0.377, p=0.717
		Amplitude	t(15)=0.537, p=0.599	t(7)=0.399, p=0.702	t(7)=0.320, p=0.758
	Monaural Left	Latency	t(15)=0.469, p=0.646	t(7)=0.199, p=0.848	t(7)=-0.925, p=0.384
		Amplitude	t(15)= -0.296, p=0.771	t(7)=-0.306, p=0.768	t(7)=0.164, p=0.874
	Monaural Right	Latency	t(15)=0.658, p=0.521	t(7)=-0.208, p=0.841	t(7)=0.418, p=0.689
		Amplitude	t(15)=-1.067, p=0.303	t(7)= -0.297, p=0.796	t(7)= -0.078, p=0.940

### P1 component

Results showed significant main effect between groups on P1 latency (F (2, 29) =23.50, p<0.001, ŋ ^2^= 0.618). Post hoc analysis revealed the shortest latency in adults with normal hearing compared to the other two children groups, which was statistically significant (p<0.001). Though children with normal hearing had shorter latencies than children with LD, the latency difference did not reach significance (p= 0.08).

**Figure 1.**
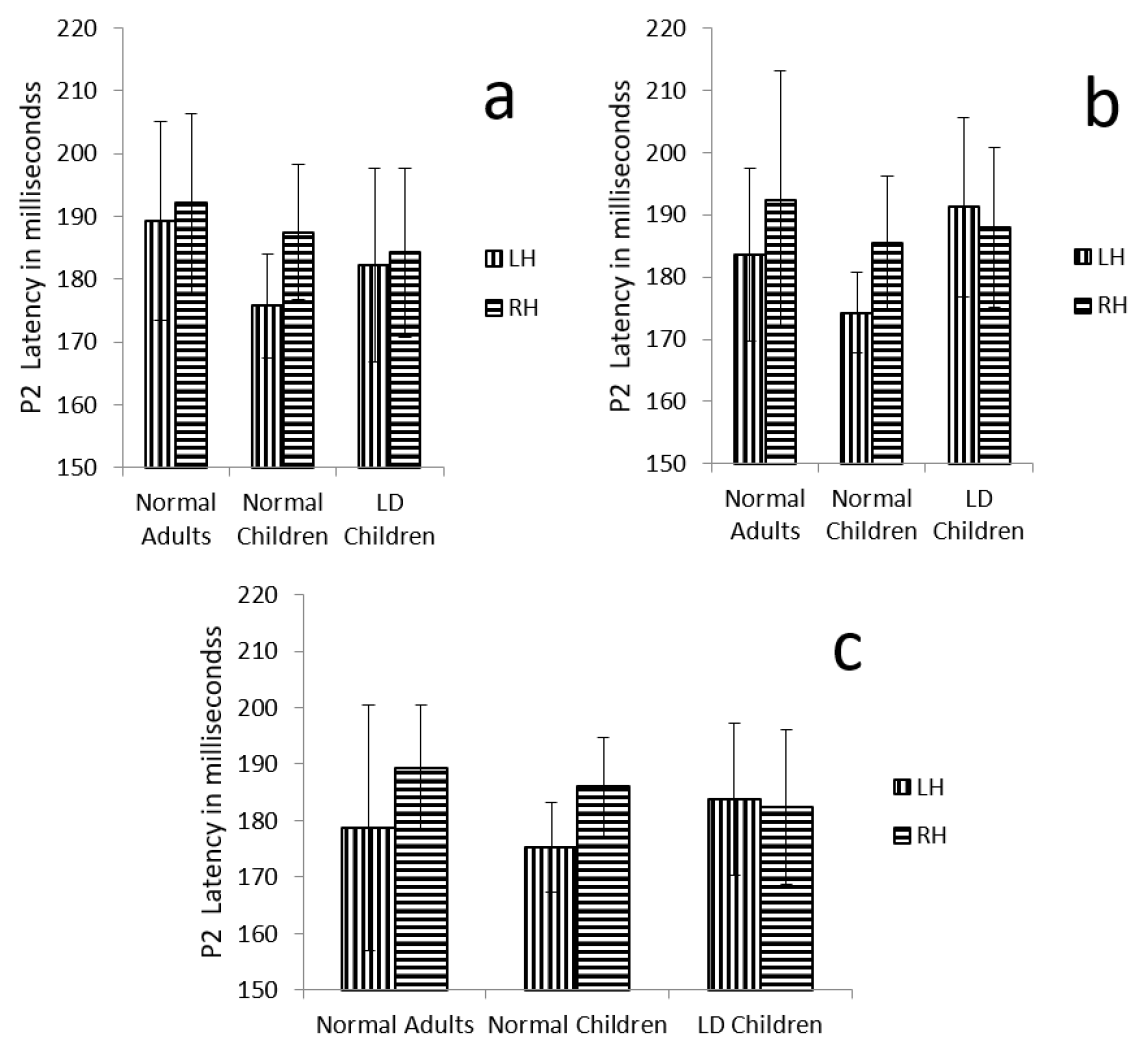
Graphical representation of P1 mean latency across ( **a**) dichotic condition ( **b**) Monaural Left conditions ( **c**) Monaural Right condition in all three groups. The error bar represents +/- standard deviation. LH – Left Hemisphere, RH – Right Hemisphere.

Further there was a significant main effect of hemisphere on P1 latency (F (1, 29) =31.8, p<0.001, ŋ ^2^= 0.523) and there was a significant interaction between hemisphere and group (F (1, 29) =3.2, p=0.04, ŋ ^2^= 0.184). These results were analyzed further by combining the condition and running a paired ‘t’ test on the data for different groups. Results showed a significantly shorter latency over the left hemisphere when compared to the right hemisphere in adults with normal hearing (p<0.001), and children with normal hearing (p<0.001). However, such hemispheric difference was not significant in children with LD (p=0.08) ( Table 2). There was no significant main effect of conditions on P1 latency (F (2, 58) =1.609, p=0.20, ŋ ^2^= 0.053) ( Figure 1 a, b and c).

**Table 2.** Mean and Standard Deviation of P1 Latency and Amplitude.

Group	Conditions	HEMISPHERE	Latency(in milliseconds)		Amplitude(in microvolt)	
			Mean	SD	Mean	SD
Normal Adults	Monaural right	LH	53.37	7.92	0.273250	0.2395121
		RH	64.7	10.5	0.118250	0.1459504
	Monaural left	LH	58.12	7.982	0.228625	0.2385755
		RH	64.75	10.580	0.114938	0.2454847
	Dichotic	LH	58	9.2	0.349875	0.1695063
		RH	63.63	5.1	0.295562	0.1470487
Normal Children	Monaural right	LH	64.00	4.781	0.223750	0.1059565
		RH	77.75	1.669	0.186250	0.2157338
	Monaural left	LH	58.12	7.982	0.141250	0.1523565
		RH	73.50	5.732	0.122500	0.1903193
	Dichotic	LH	63.63	10.889	0.301250	0.1854290
		RH	69.25	9.968	0.258750	0.2841246
LDs	Monaural right	LH	69.75	3.770	0.448750	0.8101012
		RH	72.25	6.628	0.345000	0.6979595
	Monaural left	LH	73.25	5.651	0.432875	0.6342833
		RH	76.50	7.387	0.591875	1.1768321
	Dichotic	LH	73.00	8.48	0.864375	0.8729218
		RH	74.75	9.91	1.078000	1.1805009

LH – Left Hemisphere, RH – Right Hemisphere

**Figure 2.**
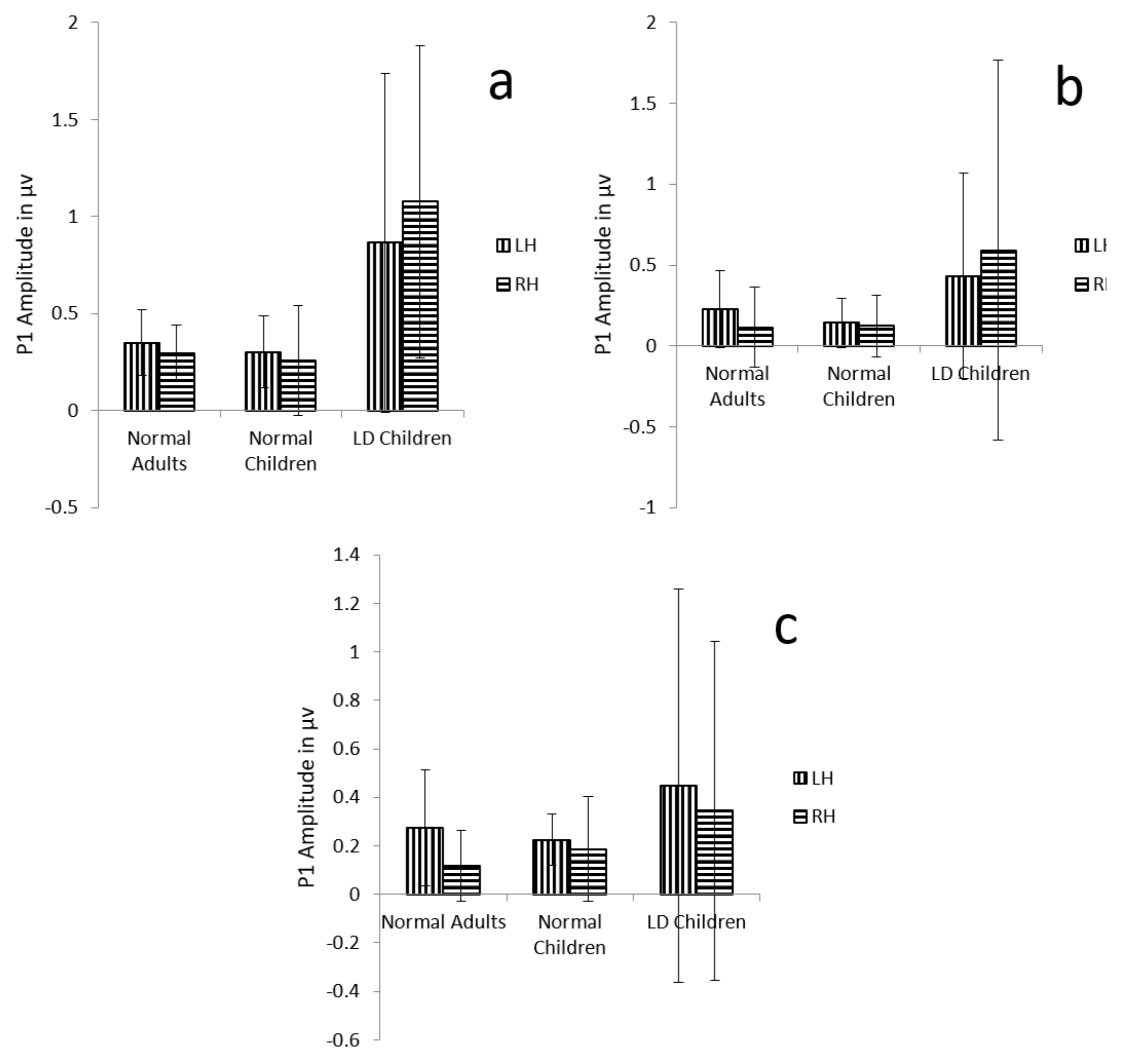
**a**) Graphical representation of P1 mean amplitude across ( **a**) dichotic condition ( **b**) Monaural Left conditions ( **c**) Monaural Right condition in all three groups. The error bar represents +/- standard deviation. LH – Left Hemisphere, RH – Right Hemisphere.

Results also revealed that there is not any main effect of either groups (F (1, 29) =1.9, p=.08, ŋ ^2^=0.12), condition (F (1.7, 51.5) =3.1, p=.06, ŋ ^2^=0.09), or hemispheres (F (1, 29) =0.049, p=0.82, ŋ ^2^=0.002) on P1 amplitude ( Table 2) ( Figure 2 a, b and c).

### N1 component

 Results showed a significant main effect of group on N1 latency (F (2, 29) =4.1, p=0.02, ŋ ^2^ =0.223). Post hoc results showed no significant difference between any of the groups (p = 0.066), though similar a developmental pattern as P1 was seen in N1 latency also.

Further there was a significant main effect of hemisphere on N1 latency too (F (1, 29) =19.2, p<0.001, ŋ ^2^ =0.399), and also a significant interaction between hemisphere and group (F (2, 29) =5.4, p=0.01, ŋ ^2^ = 0.27). These results were analyzed further by combining the condition and running a paired ‘t’ test on the data for different groups. Similar to P1, N1 latency showed a significantly shorter latency over left hemisphere when compared to right hemisphere for adults with normal hearing (p<0.001), and children with normal hearing (p<0.001). However, such hemispheric difference was not significant in children with LD (p=0.716) ( Table 3).

**Table 3.** Mean and Standard Deviation of N1 Latency and Amplitude.

Group	Conditions	Hemisphere	Latency(in milliseconds)		Amplitude(in microvolt)	
			Mean	SD	Mean	SD
Normal Adults	Monaural right	LH	104.88	10.197	-0.766688	0.5287298
		RH	119.00	10.379	-.273875	.2474962
	Monaural left	LH	106.00	11.100	-.602312	.5355764
		RH	118.38	10.614	-.541500	.3053455
	Dichotic	LH	103.38	9.344	-.663438	.5343967
		RH	114.50	8.470	-.320625	.3266515
Normal Children	Monaural right	LH	111.25	10.740	-.433750	.3364919
		RH	122.75	8.345	-.240000	.3118608
	Monaural left	LH	115.75	9.647	-.585000	.3293283
		RH	124.25	11.081	-.633750	.3781510
	Dichotic	LH	105.75	10.714	-.435000	.4411997
		RH	119.25	8.681	-.113750	.4576941
LDs	Monaural right	LH	115.75	9.647	-1.22287	.6485111
		RH	115.25	10.634	-.463125	.6199522
	Monaural left	LH	120.50	6.568	-1.01687	.7616256
		RH	120.75	9.677	-1.03287	1.3203183
	Dichotic	LH	115.00	10.085	-.916875	.8203040
		RH	113.50	8.928	-.825375	1.5277259

LH – Left Hemisphere, RH – Right Hemisphere

**Figure 3.**
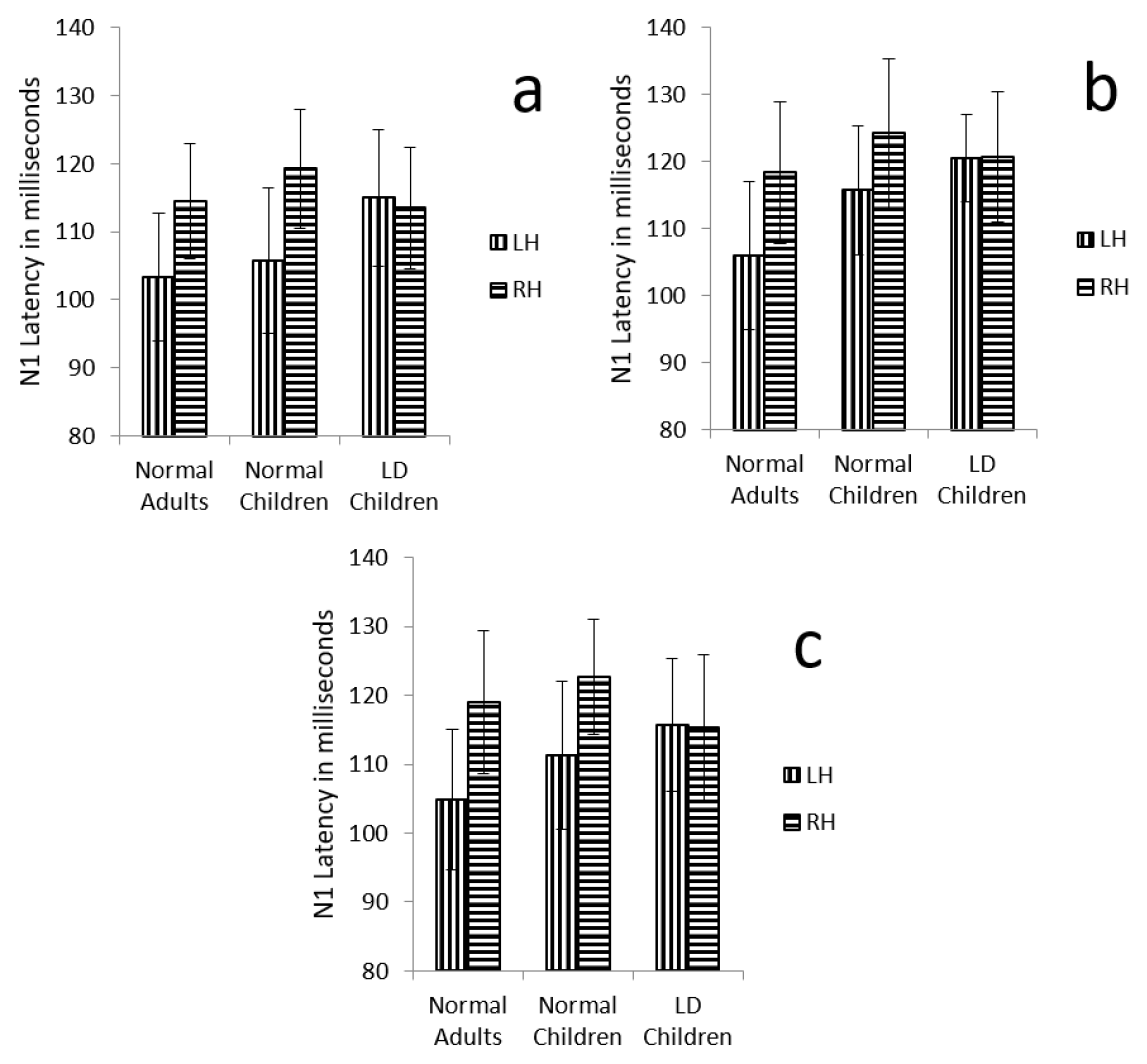
Graphical representation of N1 mean latency across ( **a**) dichotic condition ( **b**) Monaural Left conditions ( **c**) Monaural Right condition in all three groups. The error bar represents +/- standard deviation. LH – Left Hemisphere, RH – Right Hemisphere.

There was also a significant main effect of condition on N1 Latency (F (2, 58) =5.9, p=0.04, ŋ ^2^ =0.16). Post hoc results showed significant latency difference between dichotic and monaural left condition (p= 0.04) were N1 latency was shorter in dichotic condition compared to the monoaural left condition. Such significance was not seen in any of other combinations (DI vs. MR and ML vs. MR) ( Figure 3 a, b and c).

**Figure 4.**
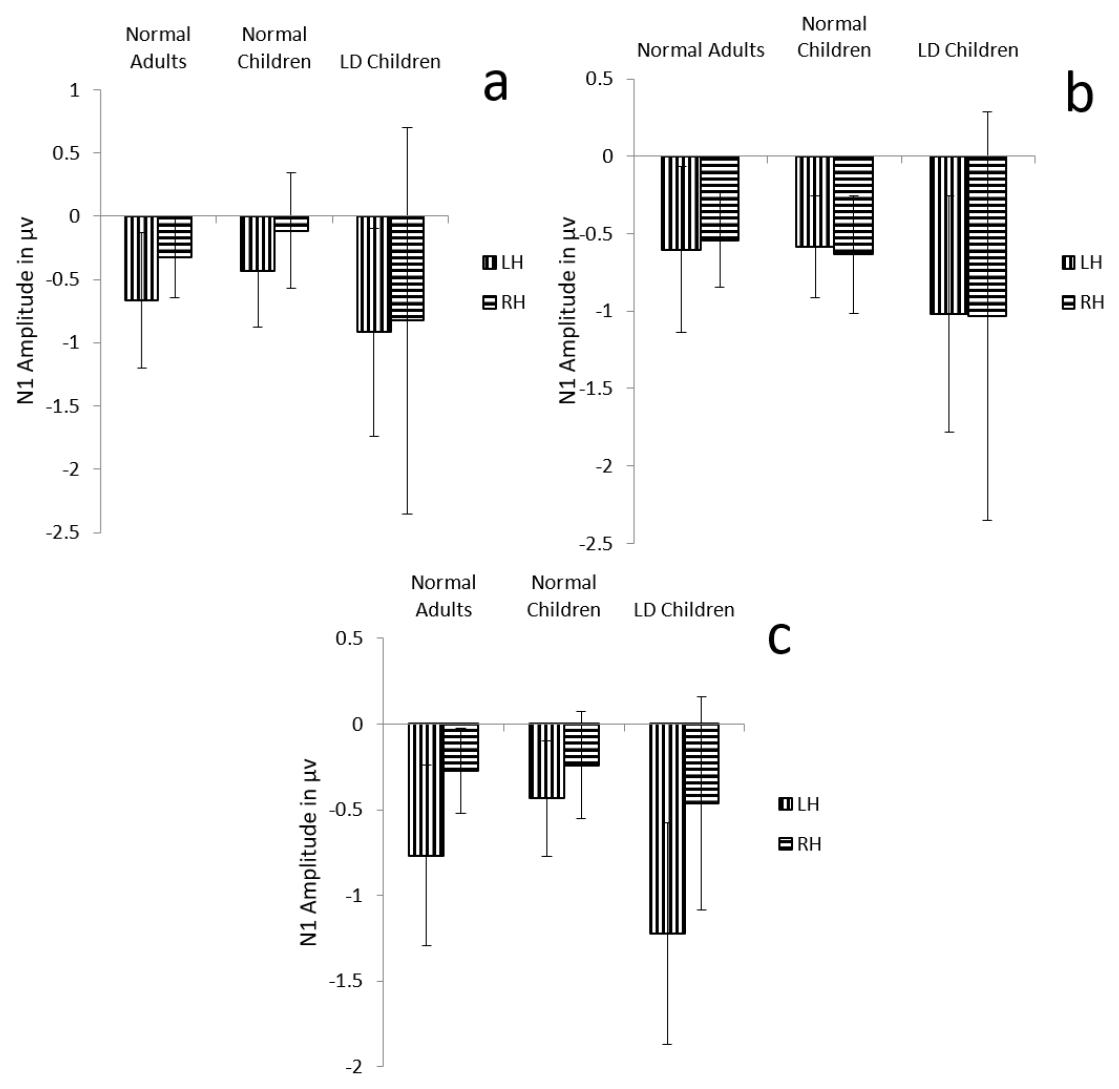
Graphical representation of N1 means amplitude across ( **a**) dichotic condition ( **b**) Monaural Left conditions ( **c**) Monaural Right condition in all three groups. The error bar represents +/- standard deviation. LH – Left Hemisphere, RH – Right Hemisphere.

Results showed no significant main effect of either group (F (1, 29) =2.8, p=0.07, ŋ ^2^=0.162) or condition (F (1.5, 44.4) =2.1, p=0.132, ŋ ^2^=0.06) on N1 amplitude. But there was a significant main effect of hemisphere on N1 amplitude (F (1, 29) =11.2, p=0.002, ŋ ^2^=0.276), and also there was significant interaction between condition and hemisphere (F (1.9, 56.1) =8.5, p=0.001, ŋ ^2^= 0.227). Further analysis revealed significantly larger amplitude over the left hemisphere in both the dichotic and monaural right condition (p<0.001), and no such latency difference in the monaural left condition (p=0.893) ( Table 3) ( Figure 4 a, b and c).

### P2 component

 Results showed no significant main effect of either group (F (2, 29) = 3.2, p=0.053, ŋ ^2^ =0.184), or condition (F (2, 58) = 0.79, p=0.42, ŋ ^2^ =0.027) on P2 latency.

**Figure 5.**
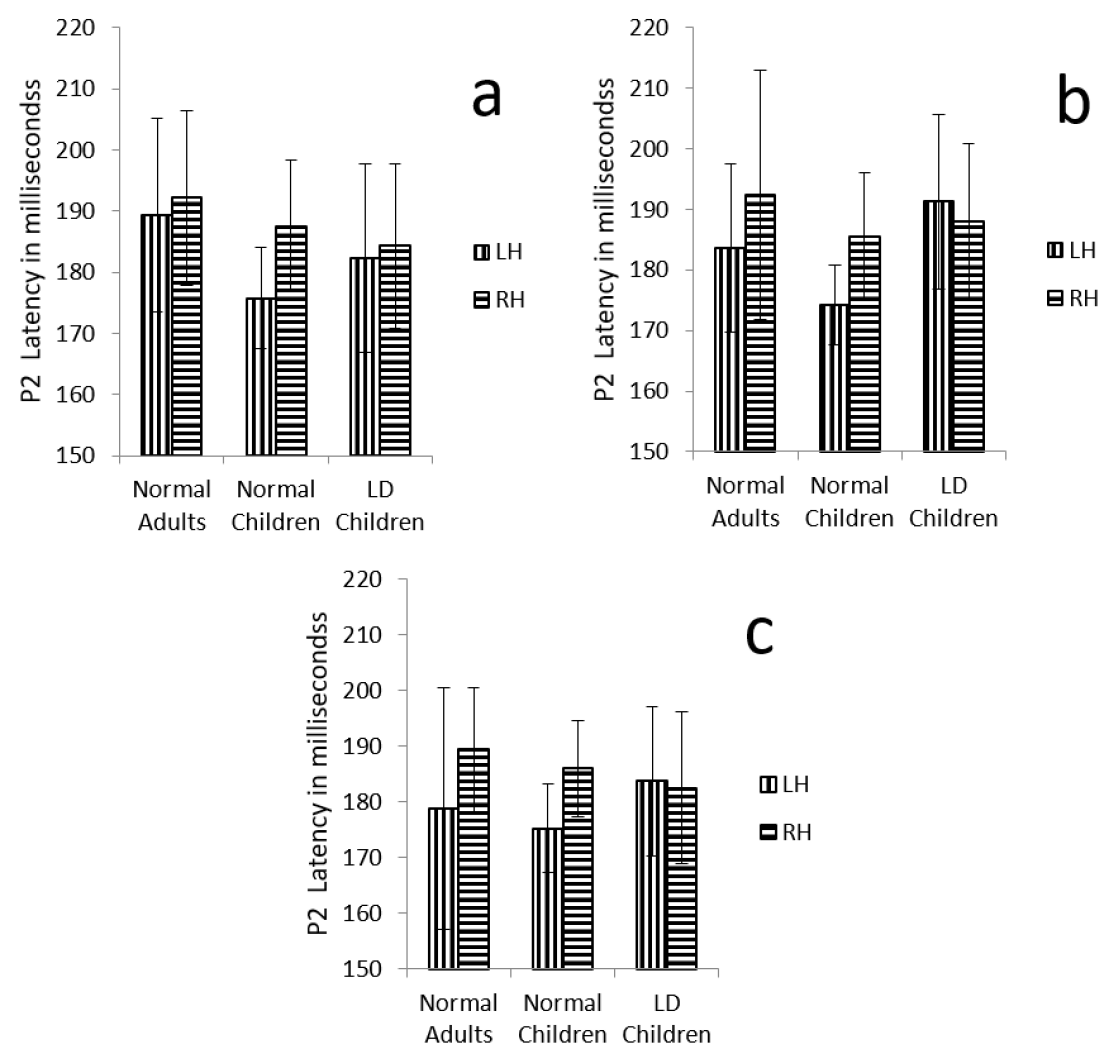
Graphical representation of P2 mean latency across ( **a**) dichotic condition ( **b**) Monaural Left conditions ( **c**) Monaural Right condition in all three groups. The error bar represents +/- standard deviation. LH – Left Hemisphere, RH – Right Hemisphere.

Further, there was a significant main effect of hemispheres on P2 latency (F (1, 29) = 4.2, p=0.04, ŋ ^2^ =0.28), and there was no significant interaction between hemisphere and group (F (1, 29) = 1.309, p=0.286, ŋ ^2^ =0.083) ( Table 4) ( Figure 5 a, b and c).

**Table 4.** Mean and Standard Deviation of P2 Latency and Amplitude.

Group	Conditions	Hemisphere	Latency(in milliseconds)		Amplitude(in microvolt)	
			Mean	SD	Mean	SD
Normal Adults	Monaural right	LH	178.75	21.663	.354500	.2453096
		RH	189.38	11.189	.196875	.2130446
	Monaural left	LH	183.63	13.937	.287875	.1861655
		RH	192.38	20.659	.143875	.1596713
	Dichotic	LH	189.38	15.828	.482750	.3126850
		RH	192.12	14.338	.238937	.2055099
Normal Children	Monaural right	LH	175.25	7.996	.217500	.1900188
		RH	186.00	8.685	.140000	.4589429
	Monaural left	LH	174.25	6.541	.427500	.3143588
		RH	185.50	10.623	.252500	.3440826
	Dichotic	LH	175.75	8.242	.330000	.1808709
		RH	187.50	10.730	.148750	.1793988
LDs	Monaural right	LH	183.75	13.414	.581750	.8795953
		RH	182.50	13.680	.461625	.8612738
	Monaural left	LH	191.25	14.459	.410250	.8326422
		RH	188.00	12.829	.396625	.7828565
	Dichotic	LH	182.25	15.471	.647000	1.5321116
		RH	184.25	13.371	.758625	1.0994058

LH – Left Hemisphere, RH – Right Hemisphere

**Figure 6.**
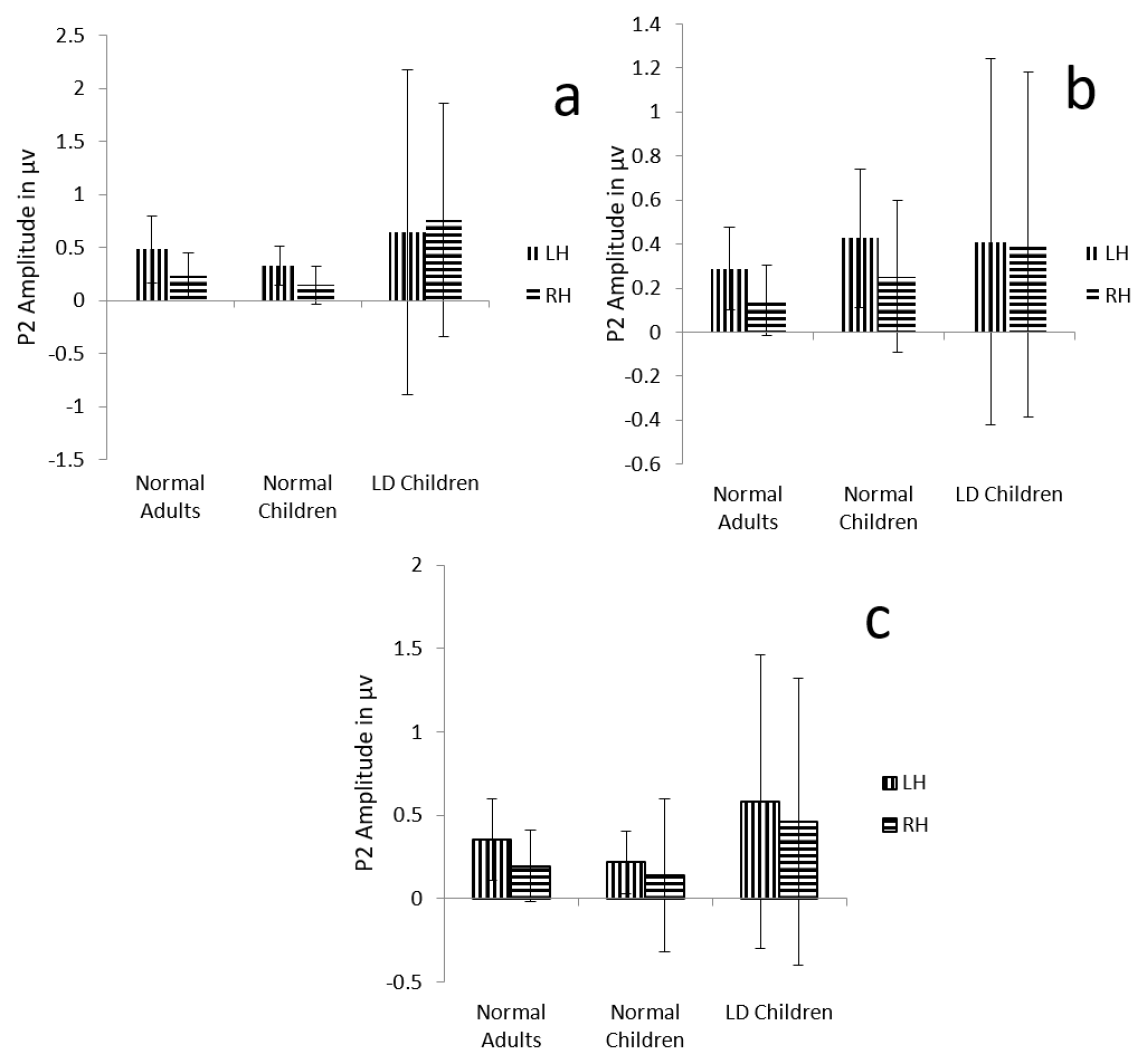
Graphical representation of P2 mean amplitude across ( **a**) dichotic condition ( **b**) Monaural Left conditions ( **c**) Monaural Right condition in all three groups. The error bar represents +/- standard deviation. LH – Left Hemisphere, RH – Right Hemisphere.

ANOVA results showed no significant main effect of either group (F (1, 29) =2.2, p=0.1.7, ŋ ^2^=0.133), hemisphere (F (1, 29) =1.42, p=0.264, ŋ ^2^=0.04) or condition (F (1.6, 46.4) =0.73, p=0.486, ŋ ^2^=0.02) on P2 amplitude ( Table 4) ( Figure 6 a, b and c).

## Discussion

This article explores the hemispheric asymmetry in three groups using CAEPs in a dichotic and monotic paradigms. While the preliminary aim of the study understands the neurophysiology of dichotic processing, the fact that monaural differences in CEAPs itself are not well understood. Hence it is worthwhile to discuss these findings in detail for the sake of better understanding of typical auditory processing.

**Figure 7.**
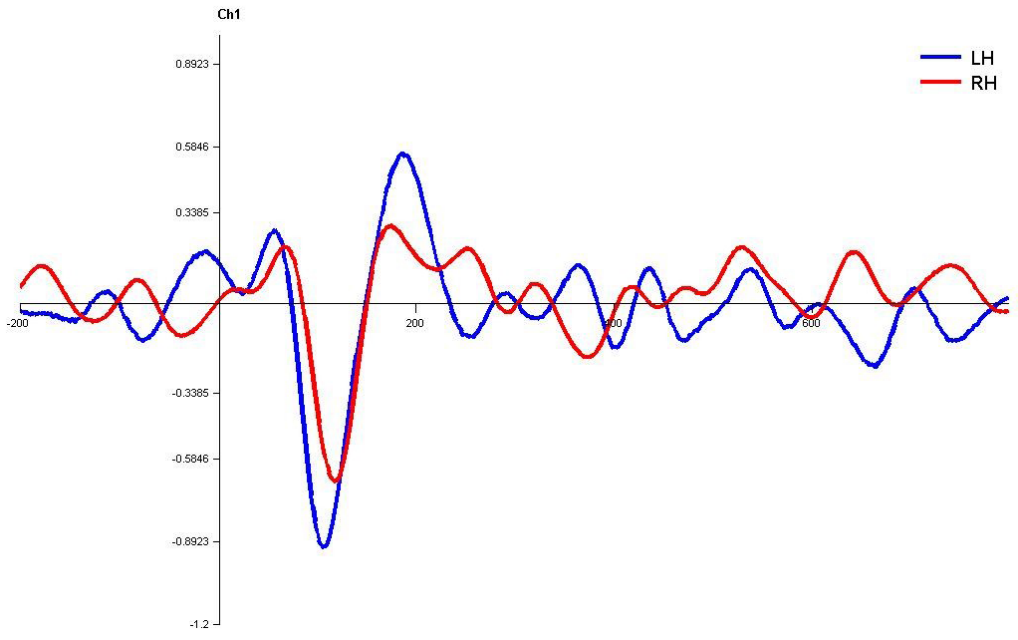
Grand average for normal adults in monaural left condition. Waveforms clearly depict shorter latency over left hemisphere than right hemisphere. LH – Left Hemisphere, RH – Right Hemisphere.

**Figure 8.**
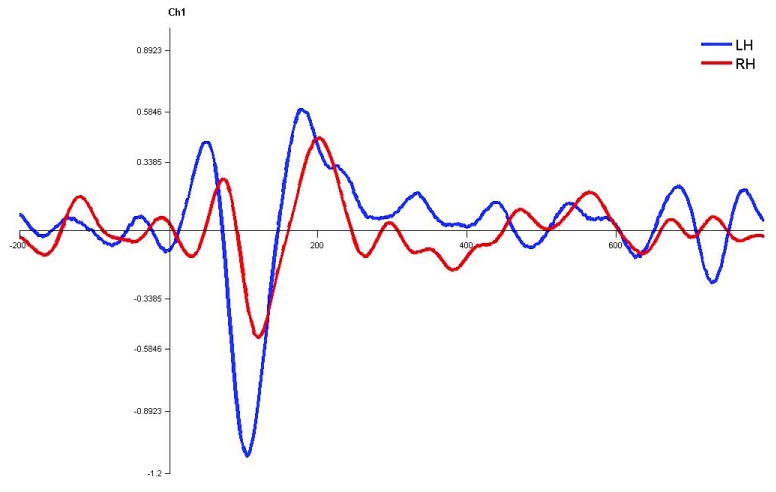
Grand average for normal adults in monaural right condition. Waveforms clearly depict shorter latency over left hemisphere than right hemisphere. LH – Left Hemisphere, RH – Right Hemisphere.

In monaural stimulus condition, the results confirmed that the stimulus effect was negligible since the latencies evoked by stops in the current study (|pa| and |ta|) were comparable. Further, it was observed that the latencies of P1, N1 and P2 components in the left hemisphere were shorter in latency compared to right irrespective of the ear stimulation ( Figure 7, Figure 8, Figure 10 and Figure 11).

**Figure 9.**
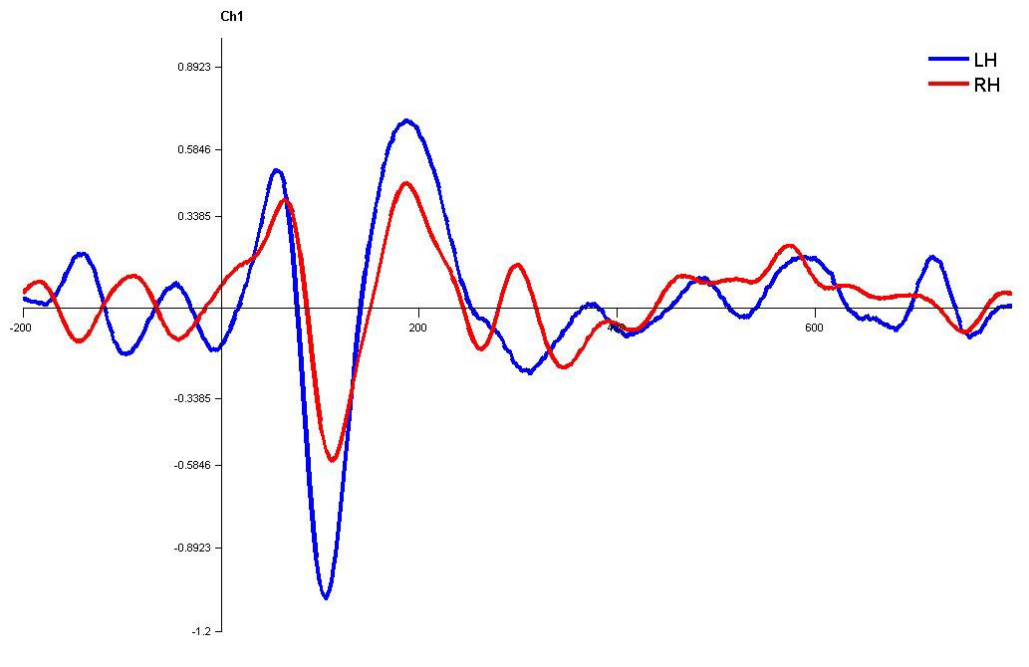
Grand average for normal adults in dichotic condition. Waveforms clearly depict shorter latency over left hemisphere than right hemisphere. LH – Left Hemisphere, RH – Right Hemisphere.

Similarly, dichotic condition resulted in insignificant order effect, and the CEAP components P1, N1,and P2 had significantly shorter latency over the left hemisphere compared to the right ( Figure 9 and Figure 12), essentially the same results as in monaural stimulus condition. It is difficult to compare earlier studies using CEAPs in dichotic listening tasks since all the CAEP components were not studied. Nevertheless, N1 latency in the left temporal electrode was shown to have 5 ms shorter latency than that of the homologues of the right ( Eichele *et al*., 2005). Another recent study reported left central electrodes were 8 ms shorter than that of the homologues of the right region ( Friedrich *et al*., 2017). They hypothesized that, under high perceptual load, the N1 predicts perceptual preferences ( Eichele *et al*., 2005). However, in the current study a similar effect was seen even in monotic listening conditions. Hence, it can be said that there could be a common perceptual preference mechanism for both monaural and dichotic listening conditions and could be interpreted in the light of hemispheric specialization for speech processing.

**Figure 10.**
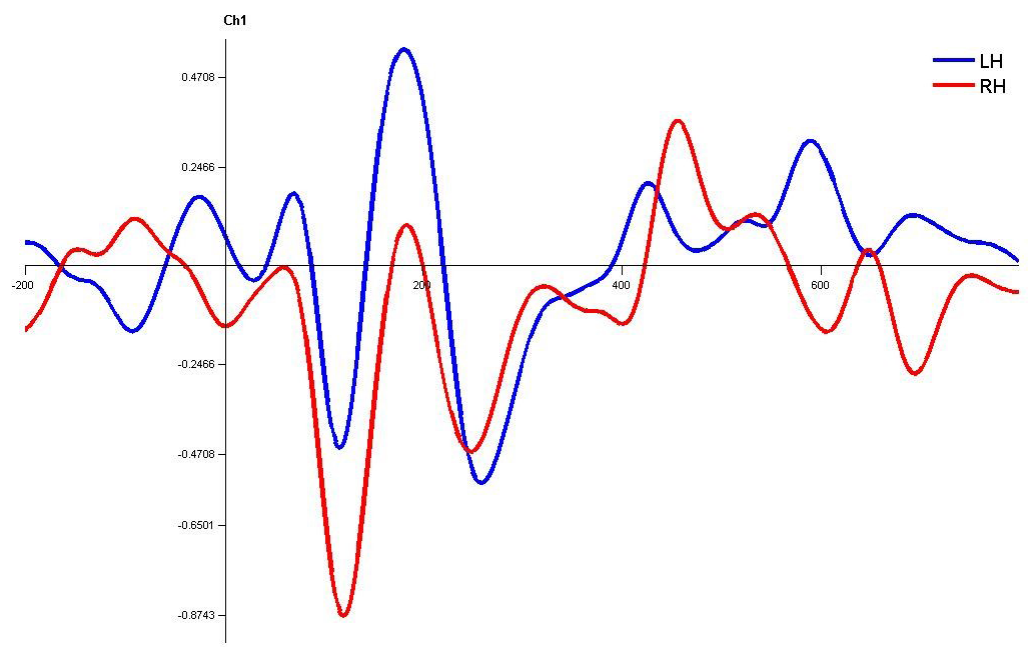
Grand average for normal children in monaural left condition. Waveforms clearly depict shorter latency over left hemisphere than right hemisphere. LH – Left Hemisphere, RH – Right Hemisphere.

**Figure 11.**
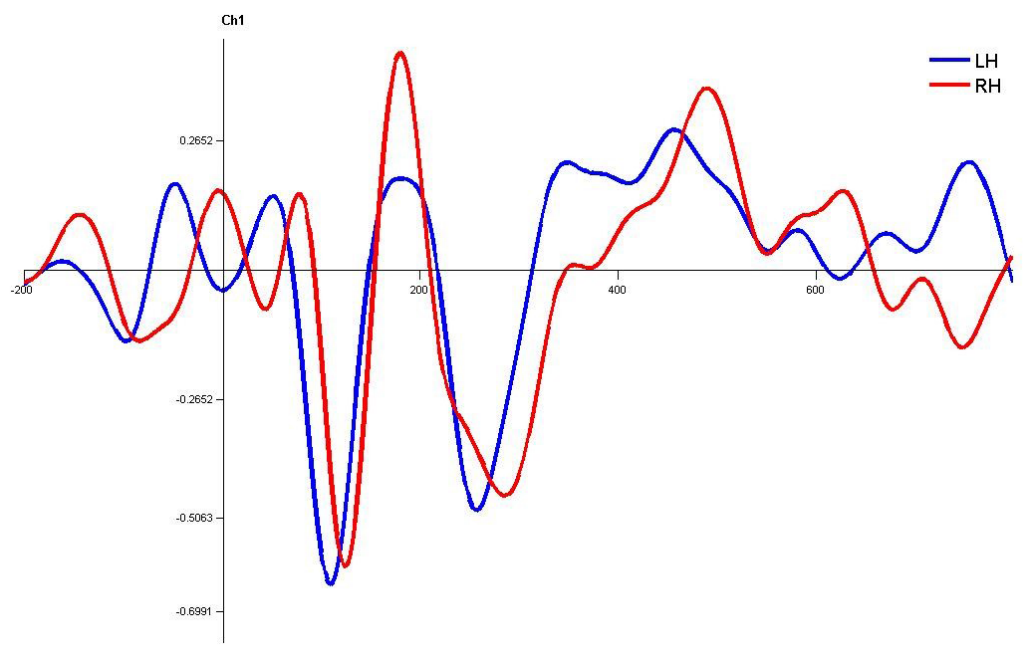
Grand average for normal children in monaural right condition. Waveforms clearly depict shorter latency over left hemisphere than right hemisphere. LH – Left Hemisphere, RH – Right Hemisphere.

**Figure 12.**
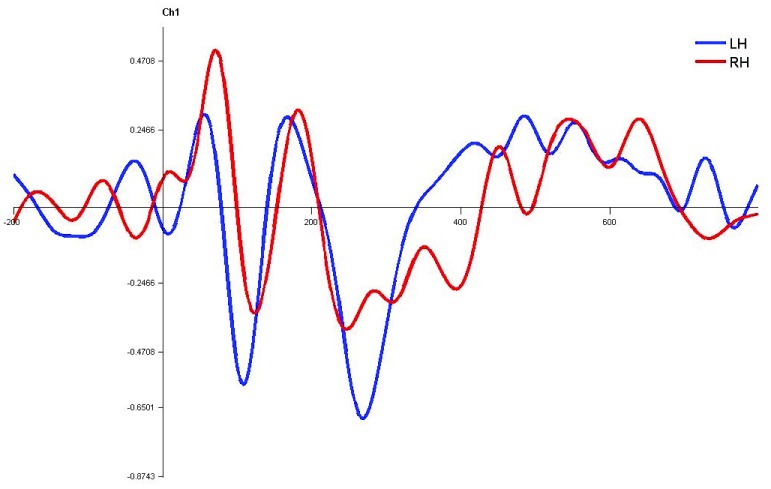
Grand average for normal children in dichotic condition. Waveforms clearly depict shorter latency over left hemisphere than right hemisphere. LH – Left Hemisphere, RH – Right Hemisphere.

Several behavioral and imaging studies in the literature have unanimously suggested that, the left hemisphere is specialized for processing speech and language related information ( Ci *et al*., 2016; Hinkley *et al*., 2016; Ishikawa *et al*., 2017; Morrell & Salamy, 1971; O’Grady *et al*., 2016; Witelson & Pallie, 1973). Though the pathway and the neural substrates are fundamentally similar, due to unknown reasons, it is proven that the left auditory cortex is characterized to have a specialized function for speech stimuli ( Corina *et al*., 1992; Witelson & Pallie, 1973).

CAEP amplitude is a variable measure as a whole ( van Hedel *et al.*, 2007). In the current study, P1 and P2 amplitude in monaural conditions were larger over the left hemisphere when compared to the right. Similar results were seen for N1 amplitude too, except in the monaural left condition. In the dichotic condition, all the CAEP components showed larger amplitude over the left hemisphere than right. Previous studies done using structured magnetic resonance imaging methods has shown similar results ( Dos Santos Sequeira *et al*., 2006). However, In the current study, none of the amplitude measures reached significance, this may be due to the low sample size in the current study or due to the inherent variance of amplitude measures.

### Children with LD

**Figure 13.**
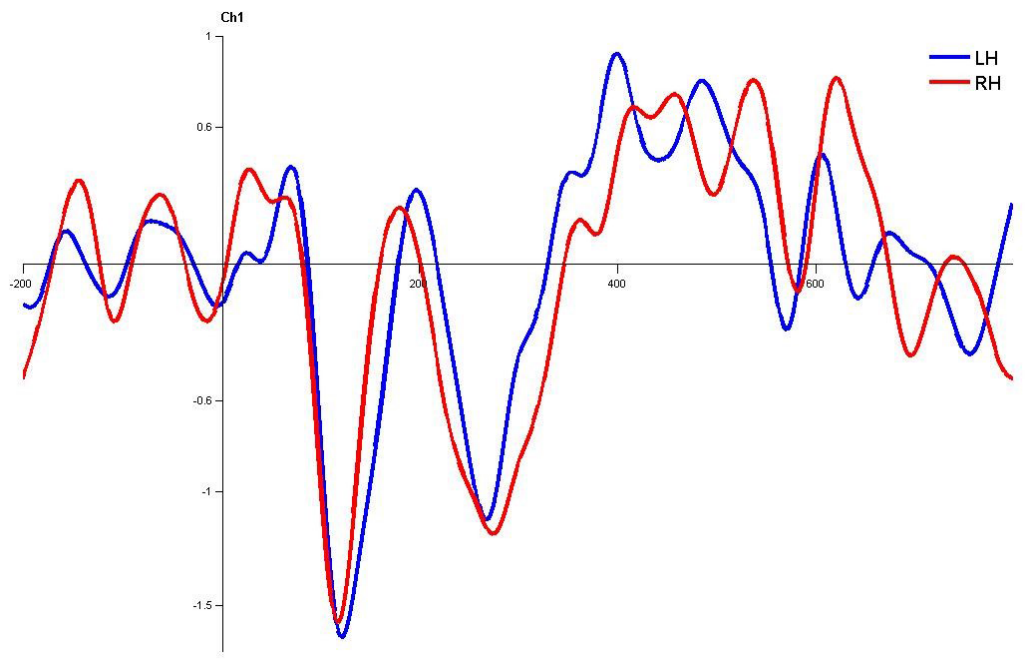
Grand average for LDs in monaural left condition. Waveforms clearly depict no significant latency difference over left hemisphere and right hemisphere. LH – Left Hemisphere, RH – Right Hemisphere.

**Figure 14.**
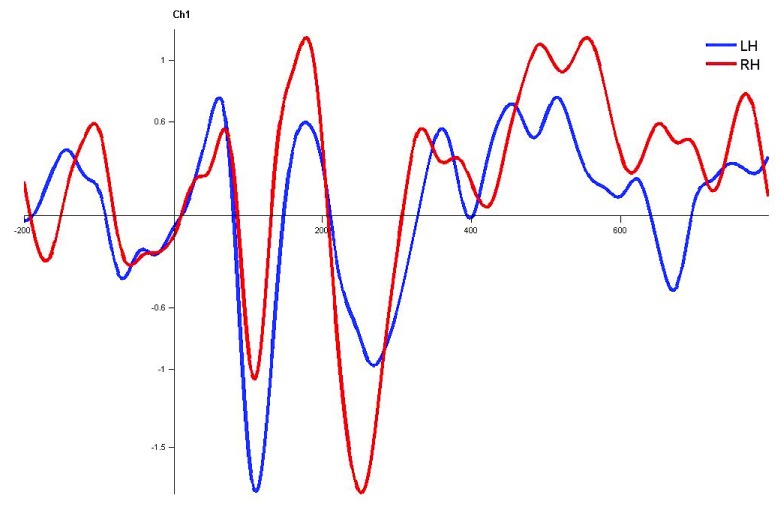
Grand average for LDs in monaural right condition. Waveforms clearly depict no significant latency difference over left hemisphere and right hemisphere. LH – Left Hemisphere, RH – Right Hemisphere.

**Figure 15.**
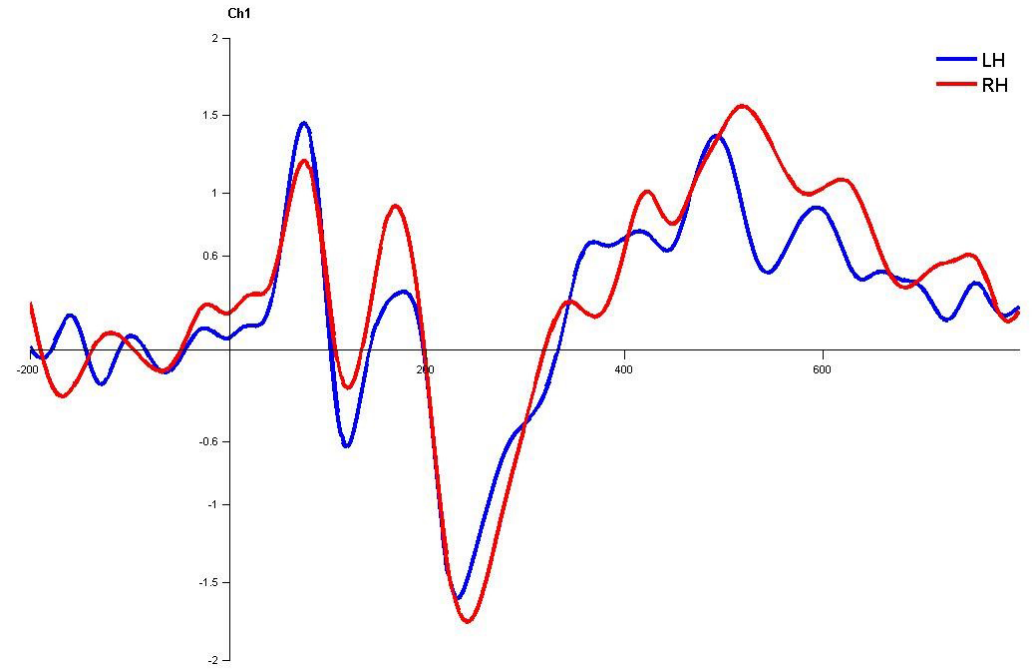
Grand average for LDs in dichotic condition. Waveforms clearly depict no significant latency difference over left hemisphere and right hemisphere. LH – Left Hemisphere, RH – Right Hemisphere

In children with LD, there was no significant latency difference between hemispheres in both monaural and dichotic stimulus conditions (Figure 4.7, 4.8 and 4.9 Figure 13, Figure 14 and Figure 15). This finding is very consistent between the P1, N1 and P2 components of CEAP. Similar findings were reported in earlier studies using neuroimaging studies on dichotic listening, where these individuals showed symmetrical activation of the bilateral auditory cortex ( Illingworth & Bishop, 2009; Njemanze, 1991). Concerning amplitude, there was no significant trend.

 In 1978, Galaburda, Geschwind, and colleagues hypothesized that patients with learning disabilities was associated with disruptions in brain asymmetry ( Galaburda *et al*., 1978). Few authors have argued that there could be atypical asymmetry in these individuals ( Cohen *et al*., 1992; Foster *et al*., 2002; Hugdahl *et al*., 1997), and others have stated that there is reduced normal asymmetry ( Martínez & Sánchez, 1999; Koltuska & Grabowska, 1975). The current study findings are in line with the latter hypothesis rather than the former. Previous studies have reported reduced cortical asymmetries in brain regions including planamtemporale asymmetry ( Foster *et al*., 2002; Galaburda *et al*., 1978; Galaburda *et al*., 1985; Leonard & Eckert 2008), corpus callosum abnormalities that of the larger total callosal areas and larger posterior (splenial) areas ( Duara *et al*., 1991), smaller anterior-most regions (genu) ( Hynd *et al*., 1995), larger posterior third of the callosum including the isthmus and splenium ( Rumsey *et al*., 1996). Apart from these, several other structures also have been reported to lack asymmetry including parietal areas ( Habib & Robichon, 1996), the posterior region of the inferior frontal gyrus ( Galaburda *et al*., 1985; Hynd *et al*., 1990), and Broca’s area ( Robichon *et al*., 2000).

Though the current study findings could easily attribute to the established anatomical deficits that are associated in children with LD, the functional asymmetry/ deficits in decoding phonological information cannot be completely ruled out. Since speech processing is well differentiated from nonspeech stimulus right from the brainstem, as earlier evidence suggest ( Abrams *et al*., 2006; Ibañez *et al.*, 1989), the observed lack of asymmetry could be a combination of both functional phonological decoding deficits as well as the structural deficits. Further, a method that elucidates the speech-specific processing from non-speech processing is at this moment warranted.

### Group effect

In the current study, there were significant differences between normal adults and individuals with LD in terms latency of CEAP components (P1 and N1), were the latencies were shortest in adults with normal hearing, shorter in children with normal hearing, and prolonged in children with LD. Previous ERP studies on these individuals have shown mixed results. Though few authors have observed no latency difference in auditory late latency response (ALLR) using click stimulus except for P1 ( Purdy *et al*., 2002), other studies suggest that individuals with LDs often have prolonged latency when compared with controls in all ALLR components ( Frizzo, 2015; Kumar & Gupta, 2014). The delay may be because of altered cortical functions ( Pinkerton *et al*., 1989), short attention span ( Picton *et al*., 1978), or deficits in auditory cortical information synchronization associated to auditory attention factors ( Leppänen & Lyytinen, 1997).

Taken together, the findings of current study, both monaural and dichotic condition elucidates the hemispheric differences in processing speech stimuli in normal hearers. At the same time, these effects are either suppressed or absent in LDs. However, there is no previous evidence to support these findings. Hence the results have to be interpreted with caution and open for exploration.

## Conclusion

The current study method is unique in comparison to previous CEAP studies, and is consistent in indicating cerebral asymmetry in normal hearers and LDs. The study failed to categorize learning disability subjects based on their specific learning disability. A lack of behavioral dichotic listening tests supplementing the electrophysiological findings can be considered as one of the major drawbacks of the current study. Overall results indicate that shorter latency and larger amplitude in the left hemisphere irrespective of the ear of presentation may indicate left hemispheric preferences for speech stimulus in normal, but lack of this difference suggests void of hemispheric asymmetry in individuals LDs. However, there is no previous evidence to support these findings. Hence the results have to be interpreted with caution and are open for exploration. Hence, based on this preliminary evidence, it can be suggested that CEAPs can be used as one of the tools to study cerebral asymmetry. Latencies of CEAP components are more sensitive to hemisphere specific difference than amplitude.

## Data availability

Underlying data is available from Figshare

Figshare: Dataset 1. P1, N1 and P2 Latency for all the group across all the condition

https://doi.org/10.6084/m9.figshare.7358387.v1 ( Palaniswamy, 2018a)

Figshare: Dataset 2. P1, N1 and P2 Amplitude for all the group across all the condition

https://doi.org/10.6084/m9.figshare.7358396.v1 ( Palaniswamy, 2018b) 

Figshare: Dataset 3. Stimulus effect and order effect for Latency and Amplitude

https://doi.org/10.6084/m9.figshare.7358417.v1 ( Palaniswamy, 2018c) 

All data is available under a CC0 1.0 Universal license

### Extended data

Stimuli used to evoke Late Latency Response

Figshare: Extended data. Stimuli used for Evoking ALLR https://doi.org/10.6084/m9.figshare.7358438.v1 ( Palaniswamy, 2018d) 

License: CC0 1.0 Universal
