# Supplementary material for: Cortical auditory evoked potentials and hemispheric specialization of speech in individuals with learning disability and healthy controls: A preliminary study
Source: F1000Res. 2018 Dec 17;7:1939. [Version 1] doi: 10.12688/f1000research.17029.1 (PMC6449798; doi:10.12688/f1000research.17029.1)
Supplement: Peer review history

# Peer review: Reviewer response for version 1

**DOI:** 10.5256/f1000research.18618.r43444

**Published:** 2019-04-03

**Authors:** Lauren Petley, David Moore

**Affiliations:** 1 Cincinnati Children's Hospital Medical Center (CCHMC), Cincinnati, OH, USA; 2 Cincinnati Children's Hospital, Cincinnati, OH, USA

**Overview**

 In this article, Bhat and colleagues aim to characterize the cortical processing of dichotically-presented speech tokens, as reflected by cortical auditory evoked potentials (CAEPs). Several stimulus- and subject-related factors are considered as possible contributors to the observed CAEP morphology: stimulus type (/pa/ or /ta/), spatial order, hemisphere of measurement, and age. To explore these factors, three experimental conditions were administered to adult and pediatric participants, consisting of a monaural right presentation of each speech sound, a monaural left presentation of each sound, and two dichotic configurations of the unmixed speech sounds. Another objective is to examine the differences in CAEP morphology between healthy individuals and those with learning disabilities. This goal is motivated by previous findings that dichotic listening is a useful test to examine cerebral dominance deficits, developmental changes in language lateralization, and bilateral hemispheric processing deficits in children with learning disabilities.

**Is the work clearly and accurately presented and does it cite the current literature?**

 There is a notable gap in the literature review for this paper regarding findings of the last ~ 15 years which have fundamentally changed scientific thinking around the cerebral lateralization of speech processing (e.g., Poeppel, 2003 ^1^, Poeppel et al., 2012 ^2^, Friederici & Alter, 2004 ^3^). The rationale for the study is generally clearly stated and the experimental conditions are logically designed to address the questions that the researchers have posed. However, the authors have failed to provide a convincing argument that the study has significant novelty or importance. Is the aim simply to fill a gap in the scientific reporting of CAEP amplitudes and latencies to dichotically-presented stimuli? Is there no greater motivation, perhaps pertaining to the clinical utility of these measures as either a diagnostic, or a method to study the neurophysiological underpinnings of learning disabilities?

 There are several easily correctable errors in this paper which confuse the reader. First, the healthy adult and pediatric participants are repeatedly referred to as “normal hearing,” but this is not a study of hearing impairment. According to the stated methodology, all participants, including those with learning disabilities, had to pass a screen for normal hearing, therefore all participants, including the disease group, must have normal hearing. Please refer to the control groups as “normal” or “controls”, not “normal hearing.” If the learning-disabled group does not have normal hearing, the methods should be updated. Second, one of the figures is incorrect: below Table 1 of P1 latencies and amplitudes, Figure 1 should depict P1 latencies, but in fact shows P2 latencies. More minor issues include: the description of the amplitude measure as “mean amplitude” in the abstract when a peak amplitude was used, the description of participant demographics in the abstract suggests that only the pediatric participants were right-handed when all participants were right-handed, frequent misspelling of the acronym CAEP as CEAP, use of the acronym LLR before it is defined, and using the word “latency” rather than “amplitude” in the final sentence of the paragraph describing N1 amplitude results. Finally, as a general comment, the paper would benefit from editing for grammatical correctness.

 The waveform figures in this paper would all benefit from considerable revision. First, none of the figures indicate which channels are represented. This can be inferred from the text but should be stated again in the figure or figure caption. The label “Ch1” above the vertical axis is particularly confusing. The units for voltage and time are also missing. Presenting each pair of waveforms in a separate, large figure makes it impossible to visually examine the effects of age, clinical group, or experimental condition. Please combine these waveforms in a way that facilitates the observation of these effects. The captions for the waveform figures are uninformative and sometimes incorrect. For example, Figure 10 states that “waveforms clearly depict shorter latency over left hemisphere than right hemisphere” when no latency differences are apparent. If anything, the latency of P1 appears to be slightly shorter in the right hemisphere. All figures, both bar graphs and waveforms, would benefit from the addition of symbols to highlight which experimental effects reached statistical significance.

**Is the study design appropriate and is the work technically sound?**

 If the goal of this study is to support future clinical and experimental use of CAEPs to study cerebral lateralization in the processing of dichotic speech, there are two major flaws in the experimental design. First, there is no justification provided for treating the learning-disabled group as a homogeneous sample despite likely representation from a variety of subtypes (e.g., reading disabilities, attention deficit disorder, and arithmetic disabilities, among others). The importance of subtyping to improve the reproducibility of research using event-related potentials in learning-disabled children has long been acknowledged (e.g., Dool et al. 1993 ^4^).

 Second, by failing to collect behavioral dichotic listening data from these participants, the authors provide no link between clinical and experimental research using behavioral and CAEP-based measurements. This is particularly important because behavioral dichotic listening paradigms do not simply reflect cerebral lateralization in speech processing, but also the influence of selective attention (Hugdahl, 2011 ^5^). The present study uses passive stimulation, except perhaps in the dichotic condition where it appears that attention deployment has not been controlled. Without knowing how the obtained CAEP measures relate to behavioral dichotic listening performance, it is impossible for clinicians or experimental scientists to weigh the relative merits of the two approaches. The purpose of highlighting these shortcomings is not to suggest that the paper is not worthy of publication, but rather to encourage the authors to appropriately address these issues in their introduction, discussion, and presumed future work that builds on these findings. Their brief mention in the paper’s conclusion does not constitute a sufficient acknowledgement of these important flaws.

 A stated objective of this experiment is to compare monaural vs. dichotic processing in the same individuals. However, the methods section suggests that a silent movie was presented under the monaural conditions and not the dichotic condition. Is this indeed the case? Like the monaural conditions, the dichotic condition is passive, so a diversionary task is still desirable to help control the deployment of attention. If a silent movie was not used in the dichotic condition, what was the rationale for excluding it?

 There may also be a problem with accurate measurement of CAEP peaks in this study. Automated or semi-automated methods to detect and measure CAEP peaks are commonly used and these algorithms must be supplied with search window bounds that are specified by the user. In this study, the search window bounds were selected based on visual evaluation of grand average waveforms across all participants. The selected windows for the P1, N1, and P2 components were 40 – 80, 90 – 140, and 170 – 220 ms, respectively. However, search windows that are selected based on the grand average waveform, particularly if computed across different experimental groups, may not perform well on all subjects. It is common for search window bounds to be subsequently modified to ensure that they truly encompass the peaks of interest at the individual subject level. While it is unknown whether the authors completed this type of inspection, the peak latencies that are reported in Tables 2 – 4 suggest that the selected window bounds for the P1 and P2 components may not have been adequate to detect these peaks at the individual level.

 By converting the search window bounds to z-scores on the normal distribution defined by the mean and standard deviation of the observed peak latency, one can estimate the percent of participants whose peaks might lie outside the search window. Among healthy adult participants, the upper bound of the P1 search window may have failed to capture the true peak for 7.21% and 7.49% of participants (in both cases, 1 out of 16 participants) for the monaural right and monaural left conditions, respectively, in the right hemisphere. Among healthy pediatric participants, the upper bound may have failed to capture the true P1 peak for 12.92% and 13.57% of participants (both 1 of 8 participants) for the monaural left and dichotic conditions, again both for the right hemisphere. In the monaural right condition for this group, again for the right hemisphere, the observed mean latency (77.75 ms) lies nearly upon the upper bound of the search window (80 ms) and the standard deviation (1.669 ms) is the smallest observed in the study, suggesting a possible ceiling effect imposed by the search window. Thus, *the mean latency of the P1 may have been artificially lowered in healthy individuals, specifically for measurements taken over the right hemisphere.*

 In children with learning disabilities, the upper bound of the P1 search window may have failed to capture 31.92% participants (2 of 8) in the monaural left condition, for measurements over the right hemisphere, as well as 20.33% and 29.81% of participants (1 and 2 of 8) for the left and right hemispheres, respectively, under dichotic conditions. Thus, *the mean latency for the P1 may have been underestimated across both hemispheres for participants with learning disabilities under conditions of dichotic stimulus presentation.*

 While the search window selected for the P1 appears to have ended somewhat too early, the search window for the P2 may have started too late, particularly for measurements over the left hemisphere. In healthy adults, the lower bound of the search window may have failed to capture the true component peak for 34.46%, 16.35%, and 11.15% of participants (5, 2, and 2 of 16) in the monaural left, right, and dichotic conditions, all for measurements over the left hemisphere. The lower bound of the window may also have failed to capture 14.01% (1 of 16) participants in the monaural left condition for the right hemisphere. In healthy pediatric participants, the lower bound of the search window may have failed for all measurements over the left hemisphere, for 25.46%, 25.78%, and 24.2% of participants (in all cases, 2 of 8 participants) for the monaural left, right, and dichotic conditions, respectively. Thus, *the latency of the P2 in healthy individuals may have been artificially increased, particularly for measurements over the left hemisphere.*

 For children with learning disabilities, the lower bound of the search window for P2 measurement may have failed to capture 15.15% (left hemisphere) and 18.14% (right hemisphere) of participants (in both cases, 1 of 8) in the monaural right condition. Measurement may also have been compromised over both hemispheres in the dichotic condition, in which 21.48% (left hemisphere) and 14.25% (right hemisphere) of participants, both equaling roughly 1 of 8 participants, may have had peaks lying outside the search window. Thus, *the mean latency of P2 may have been artificially increased over both hemispheres in the right monaural and dichotic experimental conditions for children with learning disabilities*.

 Overall, these statistical results suggest that the employed search window bounds may not have adequately captured the P1 and P2 components. However, there remains some question as to whether the reported search window bounds are in fact correct, due to the presence of latencies that lie outside these bounds in the datasets that are available via Figshare. Please specify whether visual inspection was performed to ensure that component peaks were accurately identified by the peak detection algorithm and ensure that the search window bounds that are reported in the methods section are correct. Finally, please indicate how many trials contributed to the averaged waveforms that were used for peak measurement, as the influence of noise on the observed peaks varies inversely with the number of trials used for averaging.

   

**Are sufficient details of methods and analysis provided to allow replication by others?**

 One of the objectives of this study is to examine the utility of CAEPs to dichotic stimuli to identify altered speech processing in children with learning disabilities. What are the clinical demographics of the participants in this group? Please specify what learning disabilities the children were diagnosed with and, if possible, what clinical criteria were used to render the diagnosis. Please also specify which frequencies were tested to screen for normal hearing status.

 It is helpful that the study stimuli are available via Figshare, but several important details of the speech stimuli are missing from the text. Please provide the durations of the speech tokens and specify how their intensities were matched to one another. It would be helpful to see the speech waveforms as a figure. Please specify the rate of stimulus presentation, how many stimulus trials were presented under each experimental condition, and how many of these trials survived data cleaning to contribute to the averaged waveforms.

 Some details of EEG data processing are erroneously described. The “Data analysis” section states that “… bad channels and bad blocks were visually inspected and interpolated …” but interpolation is a method that applies only to bad channels, not bad blocks of data. It also specifies that, “The data was then subjected to high pass filtering with a cutoff frequency of 1 kHz.” This is implausible because the data was originally acquired with a bandpass of 0 – 100 Hz. It would additionally be helpful to know how many ICA components were rejected per subject by the MARA algorithm.

**If applicable, is the statistical analysis and its interpretation appropriate?**

 The first hypothesis tested is that component amplitudes and latencies do not differ between the two speech tokens (/pa/ and /ba/) or between the two spatial orders of these stimuli under dichotic listening conditions. To test this hypothesis, component amplitudes and latencies were compared via a series of *t-*tests that is summarized in Table 1. Problematically, it is not specified anywhere which electrode was used to obtain these measurements. Furthermore, the authors were concerned with hemispheric differences in speech stimulus processing, yet this analysis does not appear to take hemispheric asymmetry into consideration. What is the reasoning for this approach?

 Following the statistical evaluation for stimulus effects, amplitudes and latencies were combined across stimuli. They were then tested by a 3 x 2 x 3 repeated measures ANOVA, but the factors for the ANOVA are not stated when the statistical approach is introduced. The type of test to be used for pairwise post-hoc statistics should also be specified here.

 The authors are to be commended for reporting means and standard deviations of component amplitudes and latencies, as well as effect sizes. These types of statistics are under-reported in the CAEP literature and doing so will benefit any research groups who wish to build on the results of this study. It is unknown to what extent the statistical results observed here might be impacted by amending the P1 and P2 search windows, if necessary.

 The N1 statistical results require clarification. The N1 latency ANOVA found a significant main effect of group which was inspected via post-hoc tests, presumably paired *t*-tests. No significant differences were found between the groups, but only one *p*-value was reported ( *p *= 0.066). Three pairwise contrasts should have been performed: healthy adults vs. healthy children, healthy adults vs. children with learning disabilities, and healthy children vs. those with learning disabilities. Was the same *p*-value obtained for all pairwise contrasts? If so, this should be clarified.

 Unlike P1 and N1, there was no significant effect of group on P2 latency ( *p* = 0.053).  Significance might be reached if the P2 search window is amended. There is also a typographical error in the *p* value for the main effect of group on P2 amplitude.

**Are all the source data underlying the results available to ensure full reproducibility?**

 Source data consisting of component latencies and amplitudes for all experimental groups across all experimental conditions are available via Figshare. However, these data tables are perplexing because many of the latencies lie beyond the reported bounds of the peak detection search windows. Were the search window bounds different than those reported?

**Are the conclusions drawn adequately supported by the results?**

 As noted previously, the discussion section of the paper needs to address the shortcomings of this study’s experimental design. The authors conclude that their results, demonstrating earlier component peak latencies over the left hemisphere than the right, are in line with known hemispheric specialization for language in healthy, normal individuals. This asymmetry was visible for both monaural and dichotic conditions of stimulus presentation and was generally observed in terms of larger component amplitudes over the left hemisphere as well. With respect to the children with learning disabilities, the authors state that their results are in line with the hypothesis that these children have reduced normal asymmetry. This conclusion does not appear to be supported by their results. No significant hemispheric latency effects were observed for the P1 or N1 in this group. Furthermore, inspection of Table 4 clearly indicates that, despite the lack of a significant interaction between hemisphere and group on P2 latency, earlier latencies in the left hemisphere were only observed in the learning disability group in the dichotic condition. Indeed, the authors later state that, “the observed lack of asymmetry could be a combination of both functional phonological decoding deficits as well as structural deficits.” The discussion should be more consistent in describing the lack of hemispheric asymmetry in CAEP latencies in this group.

We have read this submission. We believe that we have an appropriate level of expertise to confirm that it is of an acceptable scientific standard, however we have significant reservations, as outlined above.

## References

[1] PoeppelD: The analysis of speech in different temporal integration windows: cerebral lateralization as ‘asymmetric sampling in time’. *Speech Communication*.2003;41(1) : 10.1016/S0167-6393(02)00107-3 245-255 10.1016/S0167-6393(02)00107-3

[2] PoeppelDEmmoreyKHickokGPylkkänenL: Towards a new neurobiology of language. *J Neurosci*.2012;32(41) : 10.1523/JNEUROSCI.3244-12.2012 14125-31 10.1523/JNEUROSCI.3244-12.2012 23055482PMC3495005

[3] FriedericiADAlterK: Lateralization of auditory language functions: a dynamic dual pathway model. *Brain Lang*.2004;89(2) : 10.1016/S0093-934X(03)00351-1 267-76 10.1016/S0093-934X(03)00351-1 15068909

[4] DoolCStelmackRRourkeB: Event-Related Potentials in Children With Learning Disabilities. *Journal of Clinical Child Psychology*.1993;22(3) : 10.1207/s15374424jccp2203_10 387-398 10.1207/s15374424jccp2203_10

[5] HugdahlK: Fifty years of dichotic listening research - still going and going and…. *Brain Cogn*.2011;76(2) : 10.1016/j.bandc.2011.03.006 211-3 10.1016/j.bandc.2011.03.006 21470754

# Peer review: Reviewer response for version 1

**DOI:** 10.5256/f1000research.18618.r43254

**Published:** 2019-02-22

**Authors:** Hemanth Narayan Shetty

**Affiliations:** 1 Department of Audiology, All India Institute of Speech and Hearing (AIISH), Mysore, India; 2 University of Mysore, Mysore, India

The authors have assessed hemispheric asymmetry using. CAEPs. /pa/ and /ta/ stimuli in three conditions (mono R, L and dichotic (two orders)) which were used to record CAEPs on normal hearing adults, young adults children with age matched learning disabled children. All participants were right handed individuals. Results revealed a significant hemispheric asymmetry was observed in normal adults and adult children but not in LD children, irrespective of stimuli and conditions (MonL  L hemisphere/ right hemisphere; monR  R hemisphere/ left hemisphere and DI RH and DI LH).  

**Specific comments**

Introduction

 Recent studies were reviewed to strengthen their need. Research questions and hypothesis are missing. I felt purpose needs to still be strengthened on importance of CAEPs and hemispheric asymmetry. In addition, consider to mention the objectives of the study.     

Method

 Research design is missing.

 Justification for stimuli specifically for hemispheric asymmetry is needed. Authors have told that in previous studies similar stimuli were used to assess hemispheric asymmetry thus we also used. This explanation is not correct. Consider to justify.

 Is the stimuli is normalized? If yes, how have you normalized it? Since CAEP is exogenous potentials it is preferred to give acoustic characteristics of both stimuli (Spectrogram and spectra).

 These stimuli were presented at 70 dB HL. How these stimuli are calibrated? Consider to write the procedure of calibration. Authors have used high intensity to deliver the stimuli, is there any specific reason?      

 ROI is well explained.

 Ocular channel is activated? If yes, please specify. I know authors have removed bad channels and bad blocks were visually inspected. An eye blink induces artifacts and has an amplitude similar to that of a response.

Results

 Authors have used appropriate statistical analyses to prove the aim of study. A repeated measure ANOVA with between subject factors as group was used to assess hemispheric asymmetry in each component of CAEP (peak latency and amplitude). I feel it is two way repeated measure (? condition* 2 stimuli) with between subject factor as groups. Thus, I suggest authors to mention the factors (3*2*3). In P1, N1 and P2 latencies what is the post hoc test used? Sometimes authors have used condition and hemispheric asymmetry interchangeably. Consider to maintain the same term through out the manuscript. 

 Stimuli* condition result is mentioned and what about the interaction effect of stimuli* condition* group. Though main effect of ‘group’ is not significant but interaction of stimuli and condition may have an effect on group. Consider to give the result of stimuli* condition* group. In condition there was a significant difference and authors have used paired sample t test. There are three groups (normal, young adult and LD). If these three groups are considered then authors should use alpha corrections.  Anyway a significant difference has come but it is prepared to use alpha corrected.  

 In the same figures the waveforms of NH and LD for different condition is required rather than representing individually.  

 Discussion should have been in the heading of

 a) hemispheric difference on each component of CAEPs

1. Latency
2. Amplitude  

b) Between groups on on each component of CAEPs

1. Latency
2. Amplitude          

I have read this submission. I believe that I have an appropriate level of expertise to confirm that it is of an acceptable scientific standard.

# Peer review: Reviewer response for version 1

**DOI:** 10.5256/f1000research.18618.r42063

**Published:** 2019-01-29

**Authors:** Sandeep Maruthy

**Affiliations:** 1 Department of Audiology, All India Institute of Speech and Hearing, Mysuru, Karnataka, India

I am happy to have reviewed the article 'Cortical auditory evoked potentials and hemispheric specialization of speech in individuals with learning disability and healthy controls: A preliminary study'. The need for the study is well supported by the literature, authors have used cogent method and the results are reported well in light of hemispheric asymmetry in children with Learning disability. The study has the potential to make significant contributions to the understanding of hemispheric specialization in speech processing. However, I have few important concerns which I would like the authors to address, in order to bring more clarity and precision in the presentation of their findings.

**Abstract**

- It is not appropriate to state that there are no ERP studies. You may state that ‘there is dearth of studies’.
- ‘However, there is no event related potential (ERP) evidence, hence the main objective of the study is to explore hemispheric asymmetry using cortical auditory evoked potential (CAEPs) in normal hearing adults and also to compare the same in children with LD and healthy controls.’ Split this into two sentences.
- ‘main objective of the study is to explore hemispheric asymmetry using cortical auditory evoked potential (CAEPs) in normal hearing adults and also to compare the same in children with LD and healthy controls’. I am not convinced with this statement. I think the main objective of this study is to assess hemispheric specialization in LD and the compare the same with a group of control subjects.
- The stimuli are syllables not consonants. The syllables should be uniformly reported in IPA.
- P1, N1 and P2 are independent components. Do not report as ‘P1-N1-P2’.
- ‘The current study results suggest no statistically significant difference between the two stimulus in monaural condition and absence of order effect in dichotic condition’. This is in which group?
- ‘In healthy controls’, who are the healthy controls?
- Overall, the abstract needs to be rewritten with the help of an English expert.
- In Dichotic tests, the two stimuli are presented simultaneously, not independently.
- ‘CAEPs in DL is evidenced by larger amplitudes and shorter latencies over the left hemisphere’. Please specify the stimulus used in these studies (speech versus non-speech).
- Authors need to justify why the findings of independent studies on latency and amplitude cannot be taken together to understand the cortical dynamics. Why does a new study with both together need to be carried out?
- ‘Stimulus-specific’ instead of ‘stimulus specific’.

 

**Methods**

- Avoid using the term ‘healthy volunteers’.
- How did the authors ensure that the control participants did not have auditory processing deficits? In this study, it is not sufficient to ensure that they have normal hearing sensitivity.
- Syllables to be reported in IPA.
- How was LD diagnosed? Give more information about the degree of impairment.
- 6cm distance from whom?
- What does ‘Extended data’ refer to?
- What do authors mean by ‘normal native speaker’?
- Write it as ‘Speaker of Kannada’ not ‘Kannada speaker’.
- Define Kannada.
- I think the authors mean neutral tone and not ‘normal intonation’.
- Why were participants not tested with behavioural DL? If tested, they could have been sure of normal binaural integration in their control participants. Further, relating the ERP findings with behavioural DL would have shed more light into the underlying mechanisms.
- What was the final duration of the stimuli?
- Was any kind of quality judgement done for the stimuli?
- Was normalization carried out for the two stimuli to keep the intensity equivalent?    
- Consider providing waveforms of the stimuli, maybe with the time alignment as presented in the experiment.
- CEAPs to be changed to CAEPs.
- ‘A 32 channel electrode cap used with combined mastoid as reference’. Add ‘was’ in between.
- The spacing between the digit and the corresponding unit (For example, 100 Hz) is not correct in most places.
- ‘artifact rejection was also be done offline’ - please rephrase.
- ‘The stimulus was presented in 3 conditions’. Please rephrase this because you presented more than one stimulus in the third condition.
- ‘In both, the monaural conditions patient will be asked to watch a silent movie and ignore the stimulus presented to the ear.’ Needs to be changed to past-tense.
- Same comment for the next sentence.
- Why is it ‘passive attention condition’? The authors, I believe, did not have an active attention condition.
- As I understand, the stimulus paradigm has not changed the order of the stimuli but only the ear to which they were delivered. In terms of time, in dichotic condition, /pa/ and /ta/ were presented simultaneously. In such a case, it should not be termed as '.
- The paragraphs on stimulus effect and the order effect needs to be rewritten to bring better clarity to the readers.
- I suggest that the section on ‘stimulus’ be shifted before the section on ‘LLR recording’
- What was the total number of stimulus presentations?
- What was the task of the participants, particularly during dichotic listening? If they were passive, what were they doing during the recording?
- timelocked should read as ‘time-locked’.
- Low pass should read as low-pass. Similarly for high pass.
- ‘In the current study, two 2 ROIs with three electrodes in each hemisphere were selected, here in after synonymously referred to as right and left hemisphere electrodes’. Split this into two sentences.
- ‘Here in after’ should read as ‘Hereafter’.
- ‘the latency of N1 component from C3, FC3, and CP3 are 108ms, 110ms, 112ms respectively; then the left hemispheric ROI is 110ms’.  Start the sentence with an ‘if’.
- How was ‘mean amplitude’ measured?

 

**Results**

- I do understand that there was no significant difference between /pa/ and /ta/ in their latency or amplitude. That does not mean that there were no differences at all. Therefore, I am not convinced that the LLRs elicited for two different stimuli are clubbed together. This is likely to increase the variability in the data and mask subtle differences if any in the future analysis.
- I do see changes in the latency and amplitude LLR for different stimuli in the grand average waveform. Therefore, instead of comparing the mean amplitudes and mean latencies, a point-to-point comparison of the waves would have given a better idea of the differences between the stimuli.
- ‘significant main effect between groups’ should be written as ‘significant main effect of group’.
- ‘Though children with normal hearing had shorter latencies than children with LD, the latency difference did not reach significance’. Did children with LD have hearing loss? Please use the name of the groups uniformly.    
- ‘p’ is not reported uniformly. In some places the exact ‘p’ is reported while in others, it reports p as <0.01. Please bring uniformity.
- The names of groups in tables and figures are not acceptable. Participants with LD cannot be termed as ‘LDs’. Other groups cannot be termed as ‘normal groups’. 
- Figure 1 is expected to represent data of P1, but it has latency of P2.
- Giving the same data in tables as well as figures will add redundancy. Please give only one.
- The title of the figures and tables needs to be lot more descriptive and precise.
- Number of decimal points given in the table could be restricted to 2.
- Consider clubbing together all the grand average waves in one panel if the journal permits.

 

**Discussion **

- Rephrase ‘While the preliminary aim of the study understands the neurophysiology of dichotic processing, the fact that monaural differences in CEAPs itself are not well understood’.
- ‘CEAPs’ to be changed to ‘CAEPs’ - throughout the article.  

**Conclusions**

- ‘The study failed to categorize learning disability subjects based on their specific learning disability’. This was not the objective anyway and no analysis was meant to do this. Why is this coming here?
- Again, avoid using the term LDs.
- Conclusion will be better if it is precise and short. Present the most salient contributions for your research here. This can be followed by caveats and limitations.
- Please write the implications of your findings.

 

**General Comments**

- The manuscript needs a thorough editing of grammar, punctuation and spellings.

I have read this submission. I believe that I have an appropriate level of expertise to confirm that it is of an acceptable scientific standard, however I have significant reservations, as outlined above.
